# Isopod distribution and climate change

**DOI:** 10.3897/zookeys.801.23533

**Published:** 2018-12-03

**Authors:** Spyros Sfenthourakis, Elisabeth Hornung

**Affiliations:** 1 Department of Biological Sciences, University Campus, University of Cyprus, Panepistimiou Ave. 1, 2109 Aglantzia, Nicosia, Cyprus University of Cyprus Nicosia Cyprus; 2 Department of Ecology, University of Veterinary Medicine, 1077 Budapest, Rottenbiller str. 50, Hungary University of Veterinary Medicine Budapest Hungary

**Keywords:** adaptations, biogeography, community assemblage, diversity, ecology, ecomorphs, ecophysiology, Oniscidea

## Abstract

The unique properties of terrestrial isopods regarding responses to limiting factors such as drought and temperature have led to interesting distributional patterns along climatic and other environmental gradients at both species and community level. This paper will focus on the exploration of isopod distributions in evaluating climate change effects on biodiversity at different scales, geographical regions, and environments, in view of isopods’ tolerances to environmental factors, mostly humidity and temperature.

Isopod distribution is tightly connected to available habitats and habitat features at a fine spatial scale, even though different species may exhibit a variety of responses to environmental heterogeneity, reflecting the large interspecific variation within the group.

Furthermore, isopod distributions show some notable deviations from common global patterns, mainly as a result of their ecological features and evolutionary origins. Responses to human disturbance are not always traceable, but a trend towards community homogenisation is often found under strong global urbanisation processes.

In general, even though it is still not clear how predicted climate change will affect isopod distribution, there is evidence that mixed effects are to be expected, depending on the region under study.

We still lack robust and extensive analyses of isopod distributions at different scales and at different biomes, as well as applications of distribution models that might help evaluate future trends.

## Introduction

The present global distribution of terrestrial isopods is the result of historical, palaeogeographical, palaeoecological, and evolutionary processes filtered through more recent effects of climatic (mostly temperature and precipitation regimes), topographic, edaphic, and biotic (mostly vegetation providing shelter and food, and controlling microclimate) factors at different scales. Currently, humans exert strong effects on all terrestrial biomes and biota, mainly through habitat fragmentation, elimination and/or change, urbanisation and pollution. At the same time, humans also provide new kinds of anthropogenic shelter sites, which often favour habitat generalists and result in faunal homogenisation.

Some 15 years after the publication of the world list of all species of terrestrial isopods (Schmalfuss 2003, and the updated electronic version in 2004), we still lack a global scale analysis of distribution data. The list presented each species’ distribution at a relatively coarse geographical scale, mostly at country level. Several additional records and taxonomic revisions are published every year (e.g. Campos-Filho et al. 2017), so that today we can estimate a total number somewhat larger than the 3,710 valid isopod species belonging in 527 genera and 37 families given by Sfenthourakis and Taiti (2015) (as per April 2014). It is well known that terrestrial isopods have a worldwide distribution and exploit almost all kinds of terrestrial and coastal habitats, being absent only from polarregions and very high altitudes (> 4,800 m a.s.l. – see Beron 1997).

Oniscidea exhibit a unique feature among terrestrial animal taxa: it is the only monophyletic unit of relatively low taxonomic rank that has extant species representing almost all the range of evolutionary steps made during the transition from water to land. This becomes even more amazing if one also considers that the origin of the taxon is believed to be very old, possibly Palaeozoic (Broly et al. 2013a). Oldest known fossils are much younger (Cretaceous; Perrichot 2004, Broly et al. 2015b), but they belong to differentiated, fully terrestrial forms (Broly et al. 2013a). Genera like *Ligia* Fabricius and *Hemilepistus* Budde-Lund co-exist in time and probably represent the two extremes, from a primary amphibious life to a life under the harsh conditions of a desert. This fortunate fact offers vast opportunities for comparative studies in a wide range of fields, including physiology, morphology, ecology, behaviour, etc. Biogeography can also profit from this, by comparing modern distribution patterns among species and/or higher taxa with very different adaptive syndromes. Environmental change, such as habitat fragmentation, pollution, climate change, etc., may also exert varying effects on different lineages according to their position along this ‘water to land to extreme terrestrial habitats’ gradient. Furthermore, the study of Oniscidea distribution may provide crucial insight into processes and patterns pertaining to environmental change also due to the fairly well known general effects of relative humidity and temperature on these animals.

Global climate models predict precipitation pattern changes and increase in frequency and severity of droughts by the end of the 21^st^ century (IPCC 2014). Such changes are expected to impact ecosystem structure and function, especially where water availability is the major limiting factor for soil organisms. Furthermore, climatic factors modify the quality and quantity of substrate upon which microbes, a main food source for woodlice, act, bringing about further consequences on soil quality and productivity (Kumar et al. 1992), keeping in mind also that the duration of dry periods between rainfalls is a more important determinant of organic matter decomposition than total precipitation (Austin et al. 2004, Cable et al. 2008). The microbial community is also dependent on the temperature regime, and may change accordingly. Soil temperature directly influences biomass, microbial activity, and community composition with potentially interactive effects on ecosystems’ carbon balance (Wall et al. 2012). The general consensus found is that a warmer climate will increase metabolic activity of microbes as well as the rate of litter decomposition (Kirschbaum 1995).

In the present paper we try to overview the current knowledge on isopod distribution at different scales, as well as related issues such as diversity gradients or their role in invasions, etc. We make an attempt to evaluate climate change effects on isopod diversity, especially in view of their tolerance to humidity and temperature. Nevertheless, we should stress from the start that, despite a relatively large literature on this group, we are still far from a comprehensive understanding of their spatial patterns and the underlying processes. There are still many areas of the world that remain unexplored, especially in the tropics. Exact distributions remain poorly known, even in regions from where good species lists exist (with some exceptions, of course).

Taxonomic nomenclature throughout the text follows Schmalfuss (2004).

## Distribution patterns at different scales

Scale is one of the most crucial factors when it comes to distributional patterns. Environmental change too can be perceived quite differently and its effects can vary widely at different scales. Habitat fragmentation, for example, may lead to very different outcomes when it refers to deforestation within a stand of trees compared to the deforestation within a large forest or even, e.g., within the whole Amazon basin. Similarly, climate change at a global scale does not lead to directly analogous changes at local sites or within isopod micro-habitats. Therefore, it is important to study isopod responses to environmental changes at different scales regarding distribution patterns.

### The global/continental scale

Available information on terrestrial isopod distribution at a global scale is severely limited, though, due to large inequalities in research activity among different parts of the globe. We do know the general distribution of many species but at a low level of accuracy regarding range limits and site occupancy. During the last decade, at least, there is an increasing trend among isopodologists to provide such detailed information, but we are still very far away from a ‘World atlas of Oniscidea’. Nevertheless, even the coarse-grained data at hand can lead to some generalisations.

**Figure 1.**
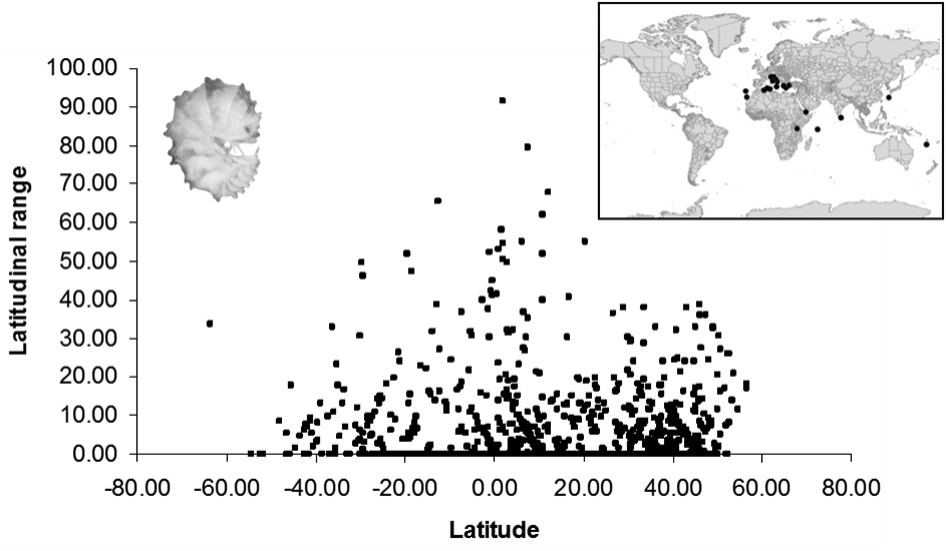
The latitudinal range of terrestrial isopod distributions is not related with latitude. The inserted map shows the diversity hotspots of endemic isopods (species with mean distributional range smaller than 1 degree of longitude and latitude) (adapted from data in Sfenthourakis et al. 2007).

Sfenthourakis et al. (2007) and Kassara (unpublished) in her diploma thesis (Universities of Crete and Patras 2006), attempted an exploration of distribution data at a global scale for the species included in Schmalfuss (2004). They used a relatively coarse-grained mapping of distributions, using a regions-by-country digital map of the world, and the information provided in the above-mentioned paper, further refined by checking the primary literature in many cases where the distribution mentioned was very vague. Specific patterns explored were diversity hotspots, latitudinal trends in species richness and Rapoport’s rule (Stevens 1989). Unequal sampling biases notwithstanding, these studies found suggestive evidence in favour of increased diversity in regions with Mediterranean-type ecosystems, as well as in insular areas of the tropics (see Fig. 1). Whether this is related to allopatric speciation patterns in topographically and/or environmentally heterogeneous sites or to other factors, it remains an open question. Latitudinal gradients of species richness are in agreement with this pattern, showing peaks in both northern and southern mid-latitudes.

Furthermore, isopods do not seem to conform to Rapoport’s rule (see Fig. 1), indicating that their distribution might not be controlled by competitive interactions, as found also in studies at local scales (e.g., Zimmer 2003). Of course, more detailed data on species occurrences and distribution ranges, especially from tropical regions, are desperately needed, before we can rigidly support any general conclusions. Nevertheless, the patterns exhibited by whole species-rich families, such as Armadillidiidae and Porcellionidae, whose global distribution is relatively well known, are highly indicative for such ‘deviating’ trends of isopods. These families are restricted in mid-latitudes and contribute to a large percentage of the global species richness, something that is not expected to change much even if groups of a more tropical ‘flavor’ (e.g., Philosciidae) prove to be much richer than it is known today.

**Figure 2.**
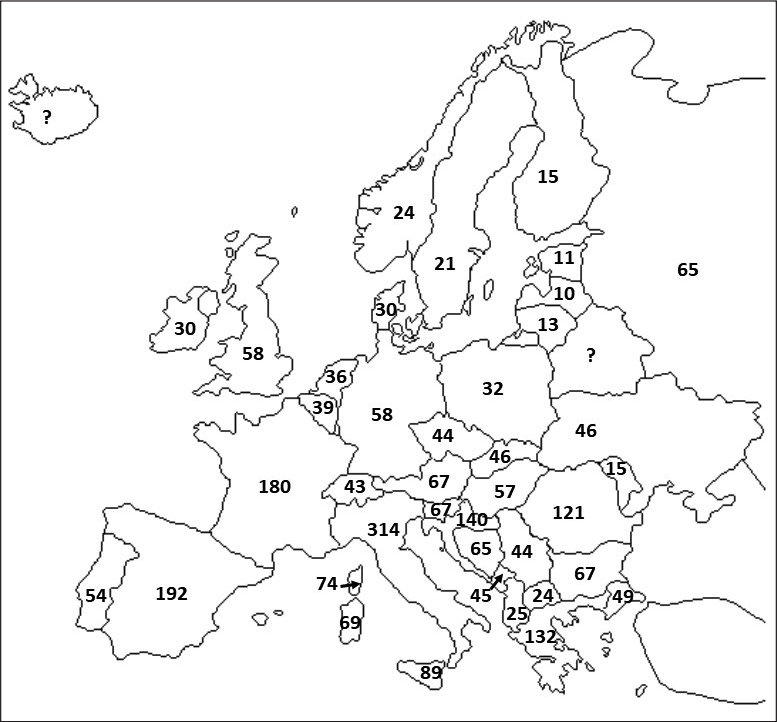
Approximated species richness for selected European countries (Corsica, Sardinia and Sicily are treated separately; Greece and Italy refer to continental parts only). Data from Fauna Europaea (de Jong et al. 2014) plus some additional country lists, corrected following Schmalfuss (2004). Exact numbers are subject to revision, but with little effect to general trend.

**Figure 3.**
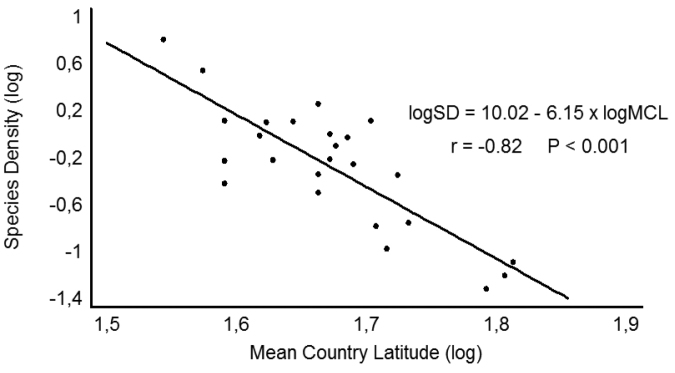
The latitudinal gradient of decreasing isopod species density (richness per unit area) with latitude among European countries (mean latitude per country). The trend remains highly significant even after the deletion of Crete and/or Sicily that exhibit very high densities. Mean country latitudes were approximated using Google Earth.

**Figure 4.**
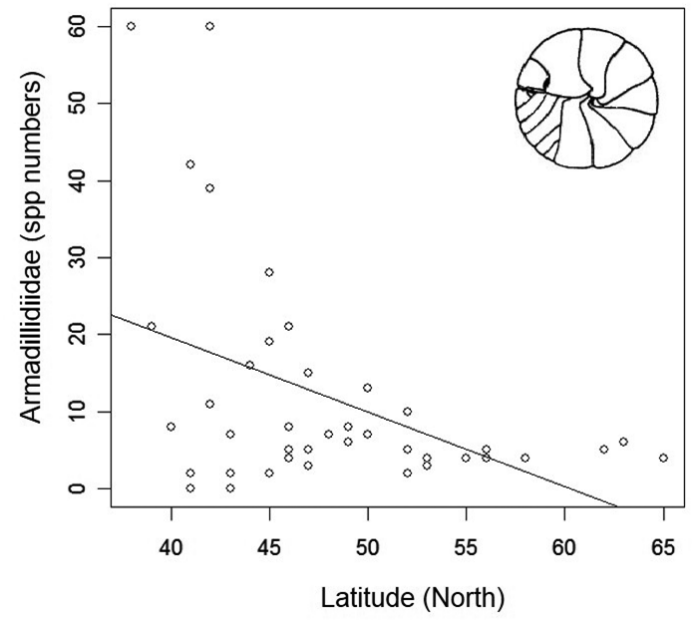
Reduction of Armadillidiidae species richness along a latitudinal gradient in Europe (the trend line of a GLM model is shown).

At a narrower latitudinal zone and an intra-continental scale, however, a significant latitudinal gradient of decreasing species richness can be seen. In fact, even though we do not have detailed maps of species richness at a fine geographical grain, when species richness of European countries (Fig. 2) are expressed per area unit (= species density), then one can find an almost perfect linear trend (in logarithmic space) of decreasing density with mean country latitude (Fig. 3). In addition, a study evaluating the latitudinal species richness of ten species-rich (with ≥ 20 species) families in Europe (e.g., Philosciidae, Oniscidae, Cylisticidae, Armadillidiidae) identified a gradual decrease of species richness towards north which was consistent in almost all families (except Cylisticidae) (Fig. 4; Hornung and Sólymos 2007). It remains to be seen whether these trends hold also true for other parts of the world. In any case, the European trend is in accordance with the tentative global trend discussed above, given that maximum isopod richness is to be found around the Mediterranean region, at low temperate latitudes.

If this pattern proves actually true and not an artefact of sampling or other biases, what would it imply for possible effects of predicted climate change on isopod distribution? On one hand, if regions around the Mediterranean become drier, thus more hostile to isopods, then we should expect a significant decrease in diversity, especially regarding locally adapted endemic forms. On the other hand, a plausible explanation for this diversity pattern could be provided by the high levels of habitat heterogeneity in Mediterranean countries, coupled with the semi-isolated and sparsely distributed favorable humid habitats, conditions that enhance allopatric differentiation hence evolutionary divergence of isopod populations. A further increase of such environmental heterogeneity through climate change might not be fatal for isopod diversity, then, but might even act as a trigger for further diversification. At the same time, positive effects could be expected for central and northern European regions, where increased rainfall and temperature might allow for range expansion of southern species, enriching diversity.

**Figure 5.**
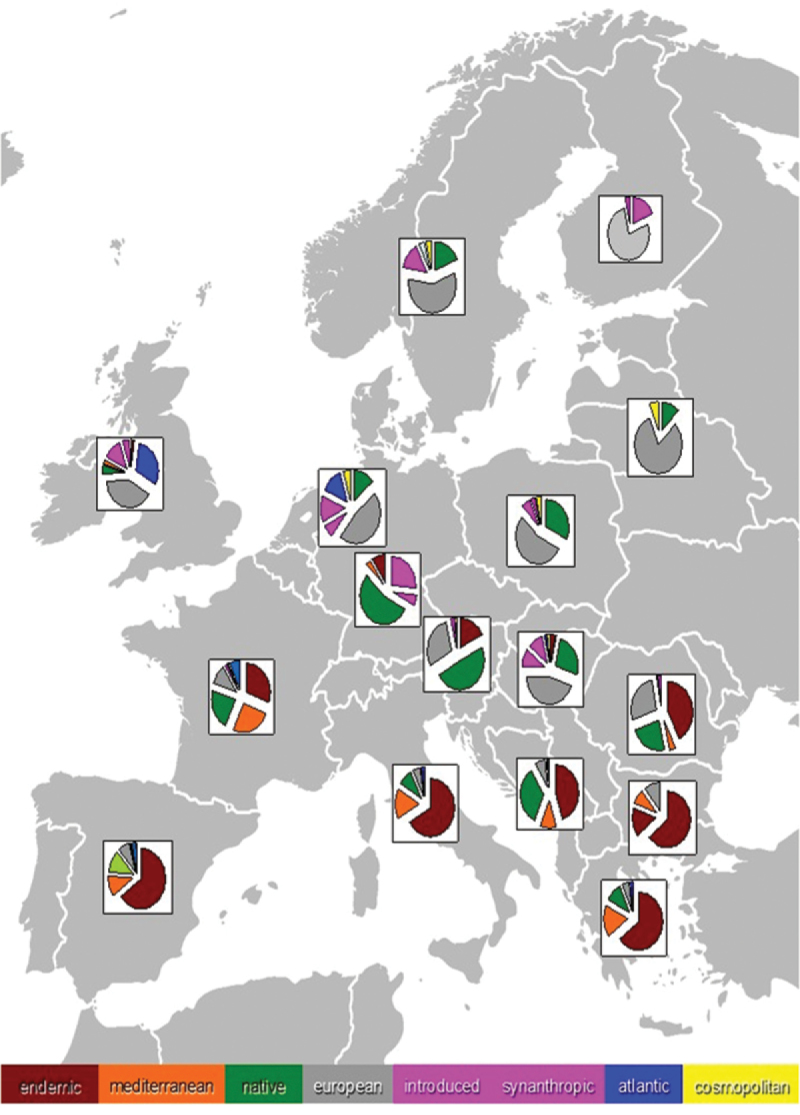
The relative contribution of different species’ categories in the isopod faunas of selected European countries.

In addition to latitudinal patterns of species richness, there is also a clear cline in the contribution of endemics and other chorological categories (Hornung and Sólymos 2007). Endemics and Mediterranean elements dominate in the south (Iberian, Apennine and Balkan peninsulas), probably because of high mountains raised barriers to northward dispersal, turning into Atlantic and European elements in the western and central Europe, even to more homogeneous, mostly synanthropic and introduced assemblages in northern Europe (Fig. 5).

A relatively good part of isopod diversity consists of troglobious species, whose distribution patterns might be particularly interesting from the environmental change point of view. In fact, it is believed that climatic changes are among the major selective stresses that lead to adaptation to a life in caves. Manicastri and Argano (1989) had attempted a global-scale analysis of troglobious species known at the time, and found again that highest diversity is concentrated in circum-Mediterranean countries and at similar latitudes in the New World (southern USA and Mexico), regions near the southern borders of glacial ice cover (Jass and Klausmeier 2006). Of course, the occurrence of troglobious species is strongly related with the availability of proper geological substrate (e.g., karstic systems), so the respective patterns might reflect the global distribution of caves instead. Furthermore, the structure of karstic systems also affects the degree of endemism, as identified by Manicastri and Argano (1989). For example, troglobious species of Greece are mostly endemic to one or a few neighbouring caves whereas local endemism is much lower for troglobionts of northern Balkans where caves form larger and more inter-connected systems. Nevertheless, this might not always be the case, as shown by the troglobious species of Romania most of which occur in a small number of neighbouring caves (Giurginca et al. 2015). Therefore, explorations on the global-scale distribution of troglobious species should be made among geologically comparable regions. A quarter of a century after this study, though, we still lack data for the isopod fauna of many cave-rich regions of the world (but see Kováč et al. 2014, Giurginca et al. 2015, Souza-Silva and Ferreira 2015, Reboleira et al. 2015), so not much can be said regarding global patterns. Terrestrial isopods, though, are among the most frequent and ubiquitous cavernicolous animal groups, even if the representation of different families in caves is highly skewed. At the same time caves provide an important control system in studies on effects of environmental changes, especially climate driven ones, so intensive studies of caves in all parts of the globe are much needed. If increased drought or lowered temperature, or a combination of these factors, has been the main selective pressure on isopods towards a cavernicolous life, it remains to be seen how expected climate change might affect them in the future, given that predictions show increased temperature and drought in many regions that are today rich in troglobious or troglophilous forms (e.g., Greece). Given that temperature in caves is usually very close to the mean temperature of their region, if troglobious species are stenothermal, we might expect increased extinction rates with increasing mean temperature, even though toglobious isopods seem to be more sensitive to humidity variation (see below). In any case, troglobious isopods can provide excellent case studies in modelling cave environments under on-going climate change. In addition, there are also other formations similar to caves, such as sinkholes in karstic regions that provide refugia at a local or regional scale. These habitats provide a ‘negative’ altitudinal gradient in temperature and humidity that effects species distribution. Rare species can be abundant in such formations, possibly able to rescue a valuable species pool under climate change (Vilisics et al. 2008, Sólymos et al. 2009).

Another attempt towards identification of global scale patterns has been made by Karagkouni et al. (2016, 2017) who explored trends in isopod body size. These authors used maximum body size of females from several hundred non-halophilous and non-troglobious species and also treated separately certain species-rich families. Body size distribution was found to be under strong phylogenetic control, Bergmann’s rule (Bergmann 1847) and the aridity hypothesis (larger sizes favored in more arid areas as a means to avoid desiccation through lower surface/volume ratio) were only weakly supported, while no support for the ‘island rule’ (Van Valen 1973) could be found. Body size (maybe also body shape; see Broly et al. 2015a) is an important factor for most biological functions (e.g., water loss, respiration, reproduction, brood size). Of course, such effects would be most important at an intraspecific level, since environmental stress towards lower size might lead to decreased reproductive success. Unfortunately, we still lack such studies that would demand exploring links among variation in body size, brood size, reproductive success etc., within the distribution range of the same species. Global trends can provide indications on the mean outcome of the effects of environmental factors on size-related features. The very restricted data at hand indicate an expected pressure towards larger size in regions where temperature and drought are increasing. Nevertheless, it is not very clear whether this means an increased extinction rate of smaller species, a higher reproductive rate of larger individuals within each species, or both. The consequences in each case might be different depending on life history strategies, position along the r-K selection gradient etc.

Based on our experience with these organisms, though, we can assume that increased drought will lead to increased extinction rates for the usually small-sized hygrophilous species through the reduction of available habitat. For example, one of us (SS) has documented the apparent extinction of *Ligidiumcycladicum* Matsakis from the island of Kythnos. This is a hygrophilous species endemic on a few Aegean islands where it lives among riparian vegetation. On Kythnos Island it was present at just one freshwater spring till 1990. After a couple of dry years in the ‘90s, the spring dried up and the species went extinct even after the re-appearance of water during subsequent rainy years. An intensification of such phenomena is expected in the near future. On the other hand, in regions where rainfall is expected to increase, the only gain in diversity might come from range shifts and long-distance dispersal (diffusion), but these are slow processes depending also on many other factors (e.g., isolation, distance to be covered etc.). It is interesting to note that this kind of observations provide a direct link between global and local-scale patterns.

### Elevational gradients

Another link between different scales can be found in the exploration of elevational gradients. These are assumed to reflect large-scale latitudinal patterns, given that changes in environmental conditions along altitudinal zones are very similar to changes encountered when one travels to higher latitudes. As already stated, isopods can be found even at very high elevations (< 4,800 m), exploiting thus a broad range of mountainous environments and providing very useful case studies on the effects of elevation gradients.

It is surprising, then, that just a handful of researchers have addressed this issue. The first such study was made on Mt. Cameroon, by Schmalfuss and Ferrara (1982), who found that isopod diversity sharply declines above the treeline. A few more studies were conducted on Greek mountains, namely by Sfenthourakis (1992) on three mountains of continental Greece, Lymberakis et al. (2003) on mountains of Crete, Sfenthourakis et al. (2005, 2012) on mountains of Peloponnisos (southern Greece) and Sfenthourakis et al. (2008) on five mountains of Greece. Most of these studies reported a general decrease of diversity with altitude (except in Sfenthourakis et al., 2005), and generally similar patterns of species richness on most mountains. The most striking common finding, though, was that on the highest elevations, above the treeline, there is usually one species that dominates in population density reaching high values of total population size. The identity of this species on each mountain-top seems to be determined by historical factors: different species of *Armadillidium* Brandt dominate on most mountain-tops of the long mountain range that is known in palaeogeography as ‘external Hellenides’ (Pindos range plus mountains in Peleponnisos and the mountains of Crete), while a species of *Porcellium* Dahl, is dominant on Mt. Olympos that does not belong to this range. This effect might be partially due to ecological release, in the absence of lizard and/or several invertebrate predators that cannot tolerate alpine environments. Nevertheless, additional factors should be responsible, given that it is exhibited by only one species even though more species often occur at the same elevational zone, albeit in much lower population sizes. It is important to note that species confined to the alpine/sub-alpine zone are very rare, and most species occupy broader elevational ranges. In Greece, at least, the only species that may be confined to high elevations is *Armadillidiumlymberakisi* Schmalfuss, Paragamian & Sfenthourakis on Lefka Ori Mt. (Crete), but even for this species there is some evidence for occurrence at lower sites (Lymberakis, personal communication). Evidence for extensive endemism of mountainous isopods, as found in Beron (1997) and Schmalfuss (2003), may not contradict the former finding, since the latter authors do not report elevational ranges of the respective endemic species. Therefore, there might be many endemics on mountains, but these may occur at several altitudes, exploiting a variety of mountainous habitats.

Lopes et al. (2005) explored gradients on elevated sites in Brazil, but the altitudinal range of their study (up to 1000 m a.s.l.) was not large enough to reveal actual effects of elevation on diversity. These authors, though, found a pattern reminding of a ‘mid-domain effect’, with species richness increasing in intermediate elevations, usually between 500–800 m. Suggestive evidence for such a phenomenon has been also found by Warburg and Hornung (1999) on similar altitudes in Israel. On the other hand, Giurginca et al. (2015) showed a decrease of cavernicolous species richness with elevation.

Most scenarios of future climate change effects on species distributions indicate a shift towards higher altitudes, especially concerning upper range limits (Parmesan 2006), particularly reducing available habitats for species restricted to mountaintops. In the face of the, admittedly restricted, information on isopods, it is not easy to make safe predictions regarding their responses to such changes, given that most species occurring on high elevations do not seem to be particularly stenoecious (i.e., with narrow tolerance regarding the range of ecological parameters). On the other hand, we might see some reduction of the high population densities of these species, especially if these densities prove to be a result of ecological release, so that more balanced communities are to be expected on high elevations. In addition, if the steep fall in isopod diversity above the tree line reported on tropical mountains (Schmalfuss and Ferrara 1982) is a general pattern, we might even expect an increase of high-altitudes’ isopod diversity following an upward shift of the tree line. The actual situation, of course, is much more complex, given the interplay of temperature rise with rainfall and humidity patterns all of which also affect isopod distributions.

### The ‘regional’ or other intermediate scales

Available information on isopod distribution at geographical scales intermediate between the global and the local are mainly in the form of country lists and, more rarely, evaluations of such lists. A seminal work in this regard is the well-known work of Vandel on the isopod fauna of France (Vandel 1960, 1962), where the author, in addition to the per species presentation of taxonomy, ecology and distribution, provided also an account of country-level general patterns. Schmölzer (1971) published a similar work for the Iberian Peninsula, but his biogeographic interpretation was rather elementary. Gruner (1966) also gave ecological and distribution data on the German isopod fauna elements. Giurginca et al. (2015), in their overview of the cavernicolous isopods of Romania, presented also a short account of the species currently known from the country.

Sutton and Harding (1989) provided a more detailed account of British isopods’ distributions, which was recently updated by Gregory (2009) taking advantage of a large number of occurrence data from Britain and Ireland. These authors showed that there is a slight northwestern to southeastern trend of increasing species richness in both England and Ireland. Despite of this trend, though, they have concluded that the most crucial factor determining species richness is habitat diversity, or better the availability of certain species-rich habitats, which tend to occur at the southeastern parts of these islands. Published work at this scale, though, is not very useful for evaluating responses to environmental factors, as they are rather phenomenological in nature, and/or mostly identify the geographical areas that host favourable habitats for isopods within the respective country or region. A more robust analysis at such a large scale was performed by Purse et al. (2012), who showed that habitat use at a fine scale is the main driver of isopod species distribution patterns.

Hornung et al. (2008) analysed distribution data from Transdanubia, the western part of Hungary that hosts the vast majority of Hungarian species, using UTM grids and recording altitude and habitat characteristics. The authors found species richness to decrease from undisturbed wet habitats to disturbed dry ones, as well as a relatively uniform richness pattern but with high compositional turnover among geographical regions and habitat characteristics. Degree of habitat degradation was identified as the main factor shaping the composition of isopod assemblages.

Another useful approach in the frame of this discussion is offered by biogeographical analyses at an intra-country spatial level, like those presented for Mediterranean island groups (Sfenthourakis 1996a, b, Gentile and Argano 2005) or the Canary Islands (Rodriguez 1991). These studies have documented in a robust way the importance of habitat diversity for isopod richness. Further analysis at the same scale (Sfenthourakis and Triantis 2009) has shown that the existence of keystone habitats, like surface freshwater sources, is crucial for the maintenance of isopod diversity. It is not the extent of such habitats that is of importance, but their mere occurrence. This suggests that if we manage to guarantee the persistence of such habitats, we can conserve most of the isopod diversity we have recorded, at least in semi-arid and arid regions.

In another approach, in an analysis of isopod community composition at different scales from local to archipelagic, Sfenthourakis et al. (2004) found that most species occurring at low abundance are local endemics, while the reverse need not be true (i.e., we can find local endemics at a high abundance). Furthermore, local endemics may be sparsely distributed in space, so that a conservation strategy based on a few biodiversity reserves may not be adequate for isopods, or even other invertebrate groups (e.g., see Sfenthourakis and Legakis 2001). These findings should alert us against an increased probability of losing local biodiversity in a wide range of areas, not only those officially designed as protected (usually based on endangered vertebrates and/or plants). It might be relevant to this discussion that the recent Red Data Book of Threatened Animals of Greece (Legakis and Maragou 2009), based on IUCN criteria, includes 47 species of terrestrial isopods whose distribution ranges hardly overlap (Sfenthourakis 2009), even if we exclude the troglobious species that are often endemic to a single cave or to a small local cave system.

Phylogeographic analyses at regional scales have started to appear during the past few years, revealing a strong effect of palaeogeographic history of current distribution of isopod taxa (Poulakakis and Sfenthourakis 2008, Kamilari et al. 2014, Lee et al. 2014) but also effects of environmental factors such as sea water temperature for amphibious species (Eberl et al. 2013) or even evolutionary and dispersal events that may lead to unexpected distribution patterns (Santamaria et al. 2013). There are still just a few such analyses so far, but we should expect a rapid raise of their publication rates given the modern advances in molecular techniques.

### The local scale – isopod communities

Community composition of a certain functional group may depend on local environmental factors and/or on dispersal limitation. Most terrestrial isopods have highly limited dispersal and dispersion abilities. Most of them are ‘prisoners’ of their special demands regarding shelters. The vast majority of ecological studies on isopods focus at the local, community-level scale. There are studies aiming to document the species composition and abundance of isopods at certain biotopes and/or to examine the temporal changes in community structure and dynamics at a seasonal or inter-annual temporal scale. It is not within the scope of this chapter to present an exhaustive review of this body of research, so we shall try to identify general patterns emerging from these studies that might be of interest for the main theme of the present topic.

Isopod communities from different parts of the world and a variety of habitat types seem to exhibit low equitability, due to dominance of one, usually widespread and/or, synanthropic species. The identity of this species, of course, may differ per geographical region and/or habitat. In Itapuã State Park, southern Brazil, for example, Almerão et al. (2006) found *Atlantosciafloridana* (Van Name) to be dominant in all transects studied, while in Europe *Armadillidiumvulgare* (Latreille) often contributes to this pattern at certain habitat types (e.g., Hornung et al. 2007, Tajovský et al. 2012). The reasons for this commonly found skewness have not been explored in detail. As already stated, this dominance pattern is very pronounced in alpine communities. At lower sites it is less extreme, but still detectable. Nevertheless, seasonal changes in dominance might also be present (e.g., Souty-Grosset et al. 2008), so the experimental design has to be taken into account before attempting generalisations.

Another aspect of isopod community structure is the moderate to high levels of nestedness exhibited among associated communities (Sfenthourakis et al. 1999, 2004, Triantis et al. 2008, Baini et al. 2014). These, nevertheless, are controlled almost completely by the inclusion of large communities in the data set, while poor communities are more or less heterogeneously structured, reflecting mostly the patchy spatial distribution of habitats. The respective literature on isopods is very restricted, though, but we can cautiously infer that oniscidean communities are principally assembled on the basis of fine-grained, local habitat heterogeneity. Along a related line of study on community assembly, Sfenthourakis et al. (2006), Pitta et al. (2012) and Baini et al. (2014) showed that there is no evidence that interspecific competition plays some significant role in isopod community structure, even among congeneric species. Once more, what repeatedly arises as an important factor across scales is the close association between isopod occurrence patterns and habitat structure (see also Hornung and Warburg 1995, Judas and Hauser 1998, Hornung et al. 2008) suggesting even a role of isopods as indicators of habitat change (e.g., Judd and Horwitz 2003).

A role of biotic interactions with other taxa in shaping isopod communities has not been conclusively shown, but some indirect evidence is suggestive. For example, Antonović et al. (2012) studying isopods in the Dubravica peat bog and surrounding forest in northwestern Croatia, found that the higher diversity observed near the bog edge could be attributed not only to edge effects, but also to predator pressure by *Myrmica* Latreille ants and lycosid spiders at the bog site.

The crucial role of key habitats for isopod communities has been shown in detail by Sfenthourakis and Triantis (2009) who identified freshwater-related habitats (riparian etc.) as the critical factor allowing for a significant representation of specialist species in Mediterranean island communities. A small core group of generalist species that forms the basis of such communities can be present even on very small dry islets, but the occurrence of freshwater-related habitats enriches insular communities with a wide range of mostly specialist species. In fact, the erratic distribution of such habitats especially on very small islets lies behind the Small Island Effect, at least for terrestrial isopods (Sfenthourakis and Triantis 2009).

Despite this strong association of isopod communities and habitat diversity, there are several studies that have failed to find significant effects of habitat disturbance on isopod diversity. Even if this sounds counter-intuitive, it is not necessarily so, as we’ll try to explain below. According to Tajovský et al. (2012), who studied isopods in 13 forest fragments in the Czech Republic ranging in area (0.1 to 254.5 ha), shape and composition of forest vegetation (thermophilous oak, mesophilous oak-hornbeam, thermophilous oak-hornbeam, acidophilous oak, basiphilous oak, beech oak-hornbeam, moist mixed deciduous forest, plantations of deciduous and coniferous trees), highest density and epigeic activity was recorded in the smallest fragments, despite of the fact that larger fragments contained a wider range of habitats. The authors found that forest fragmentation does not necessarily result in a decrease in isopod species richness. Similarly, no support for the hypothesis that diversity decreases in response to habitat disturbance or that species richness is highest in moderately disturbed sites (intermediate disturbance) was found by Hornung et al. (2007) in a study comparing urban, suburban, and rural sites in Hungary, albeit species composition did change along the gradient. According to Vilisics et al. (2007a, b), less than half of the species comprising assemblages in different habitats showed significant association with analysed habitat features. Furthermore, the responses of those that did show some association with certain habitat features were variable, reflecting their more-or-less known ecological characteristics. On the contrary, though, Messina et al. (2014) found a strong association of isopod species with specific plant communities and soil types at a coastal area of Sicily.

How can we account for this apparent contradiction regarding the role of habitats for isopod diversity? A plausible explanation lies at the scale of environmental heterogeneity exploited by such species of saprophagous animals. There is often significant variation among isopod species encountered at localities separated even by just a few tenths of meters, depending on the mere occurrence of keystone habitats, regardless of the area covered by the latter (Sfenthourakis 1994, Purse et al. 2012). This means that isopods can exploit heterogeneity at very fine scales, so that disturbance and fragmentation may not affect their species richness, as long as keystone habitats are not eliminated.

## Ecomorphological strategies and environmental change

Attention of most lay people that happen to notice terrestrial isopods is mostly attracted by conglobating species, ‘pill-bugs’, large non-conglobating forms, such as several species of *Porcellio* Latreille, *Trachelipus* Budde-Lund and *Oniscus* L., or anthropophilous common species like *Porcellionidespruinosus* (Brandt) that sometimes can be identified as belonging to the same group. It is difficult for most people, though, to see the connection between these forms and those found in Platyarthridae, Haplophthalminae, or even Ligiidae and Philosciidae. The general body design (‘bauplan’) of Oniscidea can be classified into a small number of forms, which are generally believed to be related to their ecological features. If this is true, then these forms might respond differently to environmental change. In this case, we could study responses at a more inclusive interspecific level beyond taxonomic groups.

**Figure 6.**
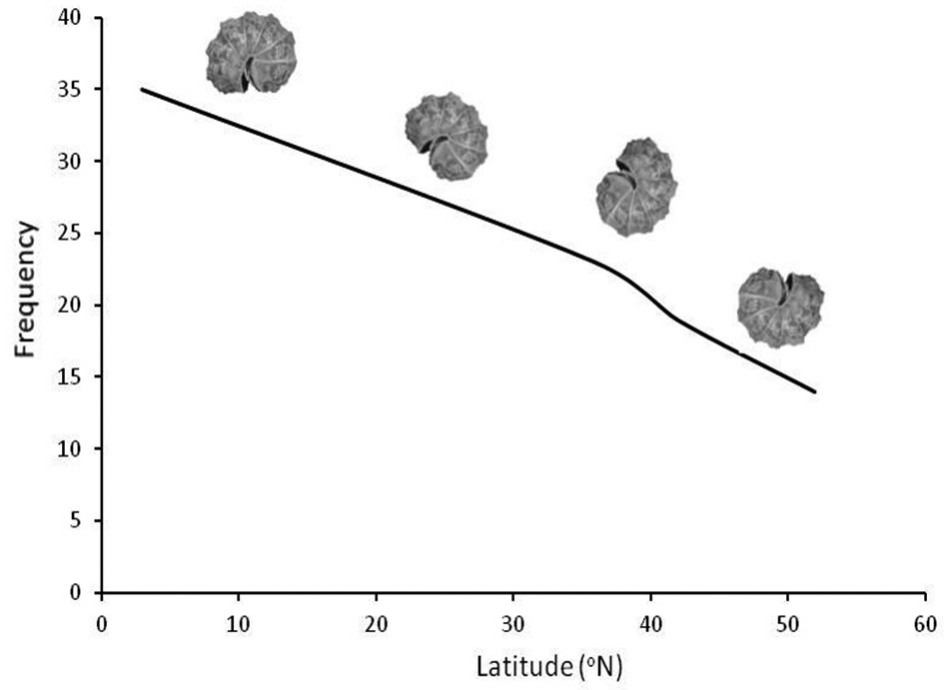
Reduction of the relative contribution of ‘rollers’ (isopods able to conglobate) among the various isopod ecomorphs with increasing latitude (based on Schmalfuss 1984).

Schmalfuss (1984) identified five ‘ecomorphological strategies’, namely rollers (able to conglobate), clingers (broad and flat bodies, attaching to the substrate), runners (long legs and narrow bodies), creepers (short appendages, body with ornamentation) and spiny forms, plus ‘non-conformists’, for species not possible to be included in one of the other five, while Hassall et al. (2006) added also ‘jumpers’ for one species of *Burmoniscus* Collinge. These are actually ‘body plans’ presumably showing responses to special ecological roles, and described the general ecology of each. The former author provided also some restricted information on possible latitudinal trends in the relative contribution of each strategy to communities from different geographic regions (see Fig. 6). Even though in the strict sense of the terms we should probably refer to these plans simply as ‘baupläne’ (‘body plans’), we shall maintain the term here to conform to isopod literature. Generalisations regarding possible responses of different ecomorphs to environmental change cannot be easily made, given a certain degree of vagueness in the current description of ecomophological strategies. In fact, Dias et al. (2013) showed that activity budgets under different relative humidity conditions were mostly related to phylogenetic relatedness than to ecomorphological strategy, since species of Oniscidae following different such strategies were predicted to be more susceptible to desiccation than members of either Porcellionidae or Amadillidiidae. Members of Oniscidae lost mass most quickly and also suffered higher mortality in drier atmosphere. We can actually assume that ‘rollers’ should be better protected from desiccation under warm and dry conditions, but this strategy seems to be related mostly to defense against predators given that it is followed by both xeric species and very hygrophilous species (e.g., several endogean, riparian or humus-living Armadillidiidae). On the other hand, ‘creepers’ seem to exploit a more narrow range of habitats compared to all other ‘strategies’, being found almost exclusively in very humid micro-sites, such as the dense plant litter-layer, wet humus and/or in caves. Therefore, a possible dramatic decrease of humidity might have negative effects for ‘creepers’ at dry regions.

A more detailed ecomorphological analysis of isopods is necessary before we can make trustworthy inferences on varying responses to environment among ecomorphs. The ‘non-conformists’ category of Schmalfuss (1984) could be divided into functional morphological categories (e.g., species of *Leptotrichus* Budde-Lund and related forms could be identified as ‘diggers’), ‘rollers’ could be further divided into, let say ‘sluggish endogeans’, ‘vigorous semi-runners’ etc. There is an increased interest in functional morphology and its relation to biodiversity dynamics in the last decade, so it is time to apply relevant approaches to this amazing group that exhibits unique morphological and ecophysiological adaptations.

## Effects of climatic factors on isopod distribution

### General

Detritivore soil invertebrates – among them terrestrial isopods – are responsible for turnover of litter and are effective regulators (litter transformers, ecosystem engineers) of that process (Lavelle 1997). The functional role of isopods is a crucial part of the soil food web. Woodlice, even though they can be omnivorous, mainly feed on decaying plant material. They have a vital role in the fragmentation, fungal and bacterial inoculation of dead plant material that is to make organic matter available for other elements of the decomposer network. In spite of the important ecosystem service of macro-detritivores in the belowground life, studies concerning their function are underrepresented in the scientific literature.

It is already widely acknowledged that climate change has altered the distribution of several invertebrate, vertebrate and plant species. Abiotic environmental factors, first of all humidity and temperature are of basic importance in the life of soil dwelling macro-detritivores, too. Climatic, microclimatic relations strongly inﬂuence the abundance, community composition and functioning of soil organisms, including woodlice, and so indirectly decomposition rates and soil macrofauna (Wardle 2002). By certain climate scenarios some soils will get wetter, others drier; the conditions and resources for soil animals are not changing consistently.

### Climatic effects

Oniscidea originate from marine ancestors and there is a distinctive gradient within the taxon of increased adaptation to land conditions so that a variety of climatic components could be identified as determinants of species’ occurrence, establishment, survival, or prosperity (Hornung 2011). In addition, the degree of dependence on such climatic factors may vary through different scales (biomes through regions, to habitats and microhabitats). In general, though, the most critical factors determining terrestrial isopod establishment, existence and survival at any given locality are temperature and humidity. Microclimatic tolerance, niche dimensions of single species or species assemblages are central topics of isopod studies since the turnout of woodlice ecology (e.g., see the works of Miller 1938, Edney 1954, 1958, Warburg 1965, 1987, Warburg et al. 1984) in the last century. Certain species are differently sensitive to environmental conditions and this broad range of evolutionary adaptation is coupled with a wide range of ecomorphological and life history strategies (Schmalfuss 1984, Sutton et al. 1984). This variety involves considerable distinct values of desiccation resistance among species (Warburg 1993, Dias et al. 2013). Protection against desiccation evolved along different routes, such as physiological, ecomorphological and/or behavioral pathways. Evolutionary changes in respiration and respiratory organs, changes in cuticle structure and morphology, conglobation mechanisms and tendency towards aggregation, are all parts of terrestrial adaptations promoting fine and large-scale dispersal (Warburg 1987, Hassall et al. 2010, Hornung 2011, Broly et al. 2013b, 2014).

Hygrokinesis and photoreaction of woodlice are of high significance to direct their reactions, movements and demographic strategies, depending on the features of the region where they occur (e.g., in temperate, xeric, mesic, semi-arid or arid environments), but there is an evolutionary trend from strongly water-dependent to more drought-tolerant species (Warburg 1968). Species differ also widely in their tolerance limits regardless of terrestrialisation stages, with habitat specialists (e.g., *Armadillidiumzenckeri* Brandt sticking to wet and cool, marshy conditions in Europe, and *Hemilepistusreaumurii* (Milne-Edwards) burrowing in loess deserts) and habitat generalists [like *Armadillidiumvulgare* or *Porcelliumcollicola* (Verhoeff)] being present in the same genera or families, and reasonably, the distribution of species along a habitat scale is in close correlation with their respective environmental requirements (Vilisics et al. 2007a, b, Hornung et al. 2008, Dias et al. 2013).

### Climate and life history

Woodlice, besides physiological and ecomorphological responses, can protect themselves from water loss by behavioural adaptations. They may show varying seasonal and diurnal patterns of activity, increased nocturnal activity and/or vertical and lateral movements towards sites with increased humidity. There is a correlation between precipitation pattern and isopod surface activity. In one study, aboveground abundance was found to increase about one month after the onset of rainy season in the Mediterranean (Warburg et al. 1984). This pattern is conspicuously reflected in seasonal surface appearance, presence or absence of woodlice in mesic and xeric regions (Davis et al. 1977, Warburg 1987, Hornung 1989, 1991, Hassall and Tuck 2007). Temperature fluctuations also can regulate population size (McQueen and Carnio 1974).

In a recent study, Dias et al. (2013) could make a generalised statement on the correspondence of water loss, desiccation resistance, and habitat choice. Based on data from 22 species, they found that differences in body water loss rate are the main mechanism behind interspeciﬁc variation in desiccation resistance. There was a low variation in lethal water loss among investigated species, while water loss rate and desiccation resistance (measured as average survival time in hours) had a significant interspecific variation in connection with ventral surface area, the main vaporiser body part. Other morphological traits such as structure of pleopodal lungs, and cuticle (Csonka et al. 2013) are also good indicators for environmental tolerance, influencing habitat choice and distribution. Regional species distribution and habitat moisture levels showed rather clear correspondence evaluating 758 records of 48 isopod species in western Hungary (Hornung et al. 2008).

Conglobating species have the advantage to reduce water loss rate and CO_2_ release: e.g. the pill-bug *Armadillidiumvulgare* was able to decrease water loss by 34.8%, and CO_2_ release by 37.1% in a relevant experiment (Smigel and Gibbs 2008). In the case of non-conglobating isopods, locomotion gives a way for active escape from predators and finding shelter, but also for avoidance of desiccation. *Porcelliolaevis* Latreille has been shown to avoid temperatures above 25 °C owing to the danger of increased evaporative water loss. There is a progressive decrease in speed after a weight loss >10 % compared to initial body mass due to desiccation (Dailey et al. 2009). Habitat specialist species are the most sensible against desiccation. For example, *Mesoniscusgraniger* (Frivaldsky), a cave dwelling isopod (adapted to a constant 93% relative humidity and around 10 °C average temperature in Baradla cave, Hungary) can tolerate gradual temperature changes on a quite broad scale (0–22 °C) but changes in humidity have a lethal effect (Gere 1970).

**Figure 7.**
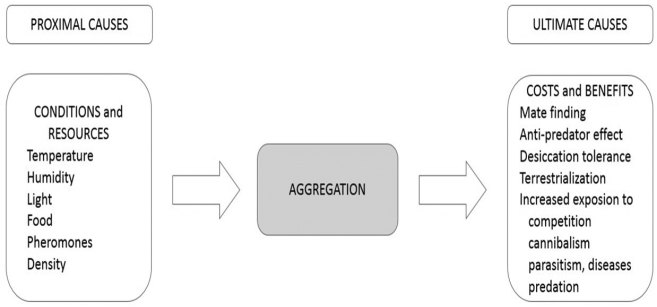
Biotic and abiotic factors (‘proximate causes’) and cost-benefit relations (‘ultimate causes’) of aggregation in terrestrial isopods as a behavioral adaptation to avoid desiccation (original idea from Broly et al. 2013b).

Another important behavioural adaptation of isopods towards water loss reduction is the tendency of many species to aggregate (Allee 1926). Water loss is dramatically reduced by crowding, as shown by several studies on aggregation patterns. Several relevant data have been summarised by Broly et al. (2012, 2013b, 2014, 2015c). Aggregation as a behavioural variation, adaptation can buffer also the effects of changes in microclimate (Hassall et al. 2010). Major proximate and ultimate causes of aggregation for woodlice are depicted in Fig. 7.

Temperature values determine also growth rate (with species-specific minima and maxima) and cohort survival rate (mancas proved to be the most sensitive). In one study, the lower lethal temperature (50% mortality) for *Porcellioscaber* Latreille was below zero, between -1.4 and -4.6 °C, with a super-cooling point at about -7 °C (Tanaka and Udawaga 1993).

Food quality and temperature have important effects on rate of gravid females and on the growth of future offspring. Females become gravid at a smaller size at increased temperature while increased food quality results in larger size (Helden and Hassall 1998).

Climatic changes definitively influence population processes, too. Life history traits such as reproduction (timing, parity, number of potential offspring) are highly influenced by humidity, moisture, and temperature (Souty-Grosset et al. 1988). There are life history parameters which help isopod establishment and range expansion. Successful species, in addition to the omnivore and detritivore feeding habits, have developed several advantageous reproductive strategies, such as parthenogenesis, multivoltine iteroparity, sperm storage, and high offspring number. Under stressful weather conditions individuals can modify their reproductive output (phenotypic plasticity) to increase survival probability (Hornung and Warburg 1998). Climate change may influence reproduction through indirect effects, such as food quality (plant growth and quality) (Rushton and Hassall 1983). The current climate change trend could affect such traits. Species with wider tolerance limits, higher reproductive output, and with the ability of sperm storage (Suzuki and Ziegler 2005) have better chances for survival and geographical expansion, and might contribute to the homogenisation of isopod fauna word-wide. Such phenomena have already been documented in urban regions, where habitat generalists and introduced, sometimes even invasive species use the human-made environment as a ‘spring-board’ for successful establishment (Garthwaite et al. 1995, Hornung and Szlavecz 2003, Tartally et al. 2004). The process is promoted by human-aided dispersal, repeated introductions, and creation of new, favourable habitats for such species. On the other hand, in species with different life history characteristics, semelparity, univoltine iteroparity, small body and brood size, and habitat specialisation hinder successful dispersal. Sutton et al. (1984) classified life history variations into two strategies, reminiscent of the extremes in the r–K selection continuum: ‘stenodynamic’, similar to r-selected, and ‘eurydynamic’, similar to K-selected life history strategies.

A great number of studies prove that there is a general relationship between female size and clutch number. Stochastic environmental changes modify the actual results on different scales (Hornung and Warburg 1998) and species show significant phenotypic variation (Dangerfield and Hassall 1992). There is intra- and interspecific, annual, and geographic variations in timing and duration of reproduction in sympatric species as well (Zimmer and Brauckmann 1997). Individuals might postpone reproduction and/or decrease output by oosorption, converting energy back when faced with extreme conditions (Hornung and Warburg 1993, 1994). Females of equal size belonging to different species may carry very contrasting offspring numbers according to idiosyncratic life histories and reproductive strategies, or to phylogenetic effects.

Despite these variations, duration of gravidity is generally temperature dependent (Snider and Shaddy 1980, Hornung and Warburg 1994). There is a threshold and an optimal range for all species. For most species, day length (and temperature above threshold values) is the trigger for oogenesis (McQueen and Carnio 1974, Hornung and Warburg 1993, 1994, 1998, Hornung 1996) and increased temperature accelerates marsupial development (Hornung and Warburg 1993, 1994). In the same species, onset of reproduction may depend also on latitudinal day-length shifts (Souty-Grosset et al. 1988).

**Figure 8.**
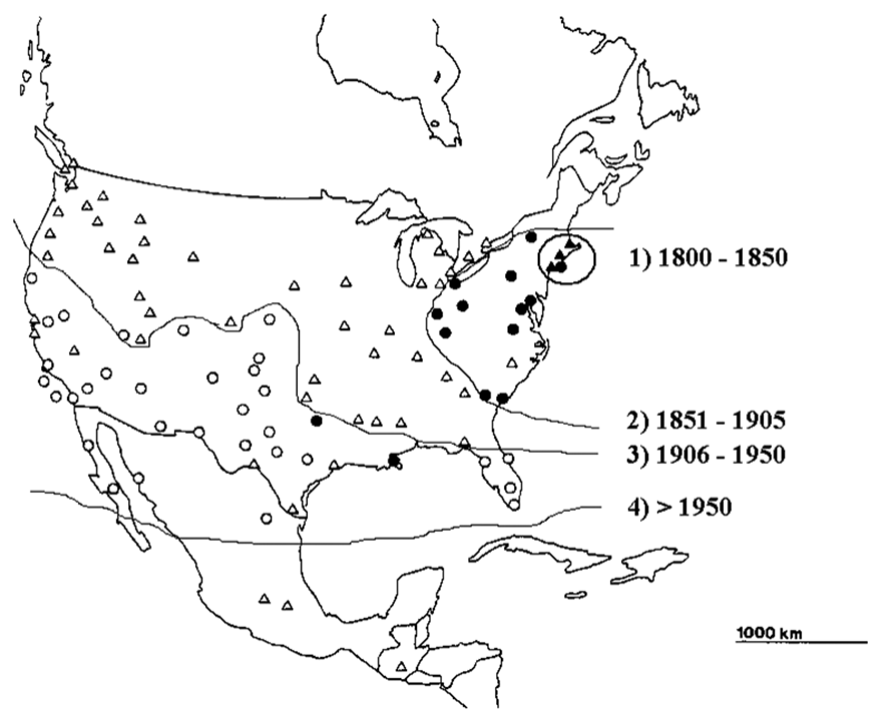
The expansion of *Armadillidiumvulgare* in North-America (modified after Garthwaite et al. 1995).

Woodlice generally are considered as having restricted dispersal abilities. Many species may hardly be able to expand their distribution ranges but some have been rather successful in colonising new territories. At the same time, distribution patterns of isopods reflect ecological tolerance at a macroecological scale. The activity radius of an individual is species specific, depending on certain ecomorphological, and life history characters, humidity requirements. *Hemilepistusreaumurii*, for example, was found to cover several meters per day while foraging (Hoffmann 1984, 1989) whilst other species, mostly small-sized, soil-dwelling ‘creepers’ (Schmalfuss 1984), move only within a few centimeters. On the other side, locomotion is essential to find food, mate, and shelter or to escape predators. In a changing environment, species are forced either to go extinct under unfavorable conditions or to migrate, following the shift of favorable environmental conditions (diffusion-type dispersal). This may have happened repeatedly in the past, e.g., during the succession of glacial and interglacial periods in the Pleistocene. In North America, the species-rich southern parts could have acted as refugia and sources for northward dispersal (Van Name 1936, Jass and Klausmeier 2000, 2006). In addition, several species were introduced from Europe, arriving in soil ballast carried by ship mainly during the 19^th^ century (Lindroth 1957), which is a jump-dispersal followed by local dispersion. Lindroth (1957) examined critically the routes of introduction of European fauna into North America, both in recent times and in the late Pleistocene. A good example is the documented historical expansion of the, originally Mediterranean but now cosmopolitan, species *Armadillidiumvulgare* (Fig. 8). Dispersal ability is the result of complex features and life-history processes. In fact, the spread of *A.vulgare* in California grassland (Paris 1963) and in North America in general (Garthwaite et al. 1995) is a real ‘success story’. Terrestrial isopods are continuously introduced in the present time. Worldwide trade of living plants, establishing botanic gardens, parks with exotic trees and so on give opportunities to introduce and spread new, mainly tropical, subtropical, or Mediterranean soil-dwelling species all around the globe (Korsós et al. 2002, Hornung and Szlavecz 2003, Hornung et al. 2005, Vilisics and Hornung 2009, Gregory 2014, 2015). In some documented cases isopod swarming was observed in different species and geographical regions. This behaviour implies occasional mass occurrence and migration of great numbers of individuals of the same. The explanation is still not clear, but should be related to demographic factors (Warburg et al. 1984). Similar phenomena occur often also in Myriapoda (Korsós 1998).

Isopods are excellent objects for anthropochorous distribution and jump dispersal. It is known that a fertilised female can reproduce several times without a successive copulation (Lueken 1963, Johnson 1982). As gravid females carry their eggs/larvae in a brood pouch till hatching, they are saved against unfavorable environmental effects. These small-sized animals can easily be propagated with soils and with ornamental plants (Hornung and Szlavecz 2003). Species introduction, a passive dispersal process today assisted mainly by humans, is usually followed by a rapid population increase and then active dispersion. Isopods might be called ‘silent invaders’: introducing a small number of specimens (in extreme cases even one gravid female) may lead to the establishment of a stable, persistent population (Vilisics and Hornung 2009). We still lack, though, a robust study on isopod propagule size, invasion dynamics, and related processes.

Interesting cases of long-distance dispersal are offered by the small and blind, specialist myrmecophilous isopod species *Platyarthrusschoblii* Budde-Lund. It probably originated from the Mediterranean region but have been successfully introduced in northern parts of Europe, accompanying *Lasiusneglectus* van Loon, Boomsma and Andrásfalvy. The ant is an aggressive, invasive species that invaded Europe (Dekoninck et al. 2007). As the ant is distributed by colony budding, isopods can easily disperse attached (Tartally et al. 2004).

**Figure 9.**
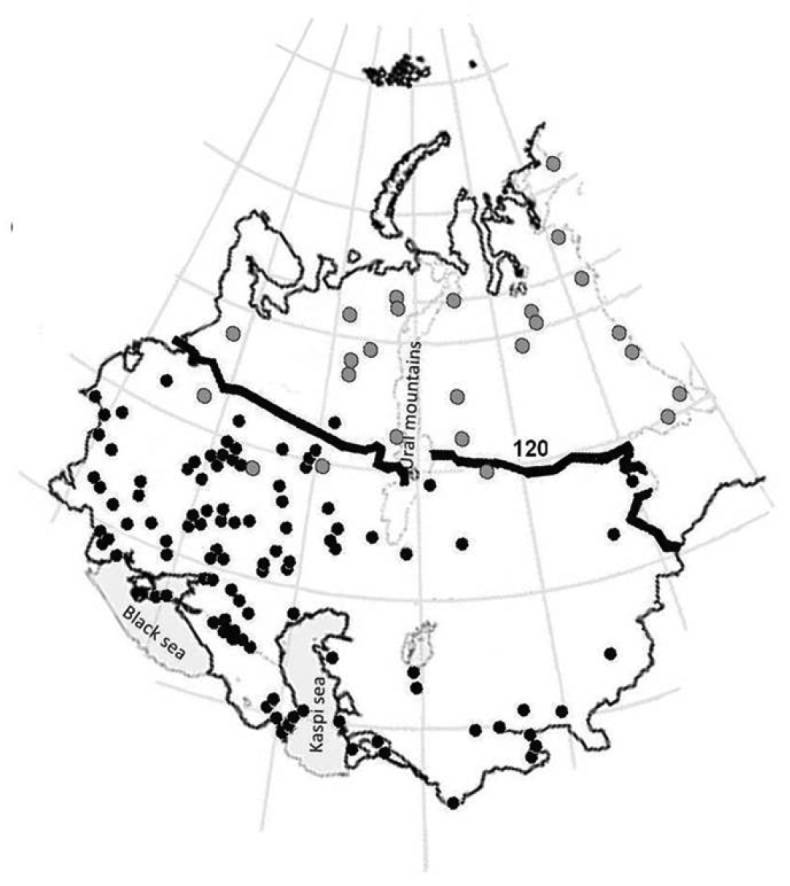
Distribution of Oniscidea in the European part of the former USSR prove the existence of climatic barrier: no woodlice were found above the line of 120 days/year with a temperature above 10 °C. Black dots – positive samples; grey dots – sample localities without isopods (modified after Kuznetsova and Gongalsky 2012).

At a larger scale, dispersal of isopods may be constrained by climatic and edaphic factors. Annual temperature, amount of daylight and precipitation regimes may condition the existence, dispersal, and activity of isopods. For example, Kuznetsova and Gongalsky (2012) found that woodlice distribution in the former USSR is thus severely constrained by the isotherm of > 10 °C for at least 120 days per year, beyond which no isopod species could be found (Fig. 9).

### Niche shift in isopods

The niche of an isopod is determined first of all by microclimatic conditions. Michael R. Warburg, as a postgraduate student of G. Evelyn Hutchinson, made the first studies on isopods’ niches. He partitioned an isopod’s niche into two parts (Warburg 1965): habitual (commonly occurring situations) and extreme (extreme situations). Nowadays, the niche might be compiled of the effective values of climate/microclimate, resources, intra- and interspecific relations, and all physical and biological factors influencing reproductive output. Very often chemical, physical characteristics of soil, such as pH and/or carbonate content are limiting factors (Zimmer et al. 2000) and ecophysiological requirements direct distribution of woodlice. The actual microclimatic changes influence the daily activity, diurnal and seasonal rhythm of the populations. Above-ground surface activity and activity density of the populations belonging to different species also depend on their affection to soil temperature, soil moisture, above ground relative humidity and availability of shelter microsites as well. Humid shelters are particularly important during reproduction, egg, embryo development, and manca release to save offspring against dehydration.

Cold adaptation might exist among isopods. *Porcellioscaber*, a species with worldwide although mainly synanthropic distribution, has proved to be tolerant of temperatures below 0 °C. Its super-cooling point was approximately -7 °C (Tanaka and Udagawa 1993). Active specimens of different woodlice species occur rather often under snow inside the decomposing litter layer in temperate regions. Such kind of abilities might help both the altitudinal and latitudinal dispersal of cold-tolerating species. The expected effect of climatic changes is the shift in distribution limits at least in the case of habitat generalist and synanthropic species. The potential ‘climate change-driven’ northward range expansion of certain terrestrial isopods (and other detritivores) might contribute to the increase of litter decomposition rates. Decomposition rate changes depend mostly not on species composition but on the biomass of macro-decomposers. Increased decomposition facilitates carbon emission (van Geffen et al. 2011). In consequence, superdominance of generalist, widely distributed species itself might cause important changes in decomposition rate and carbon release.

On the other extreme, a study on *Armadillidiumvulgare* proved that critical thermal maximum decline interrelated with declining oxygen concentration (Klok et al. 2004). A developed ‘tracheal’ system in certain terrestrial isopods, such as in *A.vulgare* (Csonka et al. 2013) makes oxygen delivery more efficient. Hypoxia causes a considerable effect decreasing metabolic rate and upper thermal tolerance, compared to insects.

Of course, other factors besides climatic ones may also constraint the ability of isopods to disperse or undergo niche shift. These involve ecophysiological requirements, soil pH, soil calcium content etc. (Zimmer et al. 2000).

## Conclusions, open questions, and future directions

Climate change has always been a trigger for evolution and for changes in the distribution of all species on Earth. Nevertheless, if one of the various scenarios of global climate change presented by IPCC proves to approximate the actual climate of the next few decades, then the speed of the change will not allow for distributional shifts and evolutionary adaptations of most organisms. Different scenarios forecast global temperature and sea-level rise, and more frequent occurrence of extreme climatic phenomena. The extent of climate change effects on different geographical regions, though, is expected to vary widely.

As far as terrestrial isopods are concerned, we still need to gather more detailed data before we can provide reliable estimates of expected changes in distribution patterns. Based on available evidence, as presented in this paper, though, we may attempt some general tentative hypotheses. Most scenarios predict increased drought in circum-Mediterranean regions, which are assumed to become even more arid than today. Since the highest known species richness of isopods is found in this region, it is very probable that a significant part of isopod diversity, primarily those more dependent on humid habitats (such as riparian etc.), will be negatively affected. Such effects will be more extensive in insular assemblages where water availability is even more restricted. Given that Mediterranean islands host many endemic species, the expected problems will be also of qualitative importance. On the other hand, higher latitudes (and, also, altitudes) may experience an increase in isopod diversity, if species will be able to expand their distribution ranges following increasing annual temperatures. Nevertheless, in most regions that will not experience pronounced habitat alterations, we do not expect to see significant effects on isopod assemblages, given that these animals are not particularly sensitive to intermediate levels of disturbance.

An important relevant process that, till today, has led to an increased isopod diversity in some regions of the world (e.g., North America, Britain), is the human-caused introduction of alien species (Jass and Klausmeier 2000, Hornung and Szlavecz 2003, Gregory 2009, 2014, 2015). Coupled with the fact that isopod species do not usually exhibit pronounced interspecific competition, introduction of alien isopod species is not expected to have negative effects on native species, so that the net result will be an increase in species richness. Nevertheless, there are some cases where introduced species may have led to exclusion of natives (Taiti, personal communication). Such effects might be seen also in isolated tropical islands and human-made ‘tropical houses’ after the anthropochorous dispersal of pantropical species, but also in temperate regions where several species have been introduced mainly of European origin. Temperature increase might provide more opportunities for alien introductions. Of course, this process has its negative side too, since we do not yet know the effects of other alien taxa introductions on native isopod species (e.g., possible predators such as spiders or lizards etc., or competitors such as millipedes, detritivore larvae etc.).

Climate change may also alter vegetation that can lead to changes in litter quality, which in turn may also influence detritivore species like isopods. The processes involved, though, are very complicated, so we cannot make safe predictions at the current state of knowledge.

Climate change, of course, is not the only important factor behind on-going environmental change. Besides chemical and organic pollution that might be important in some places, the most important processes for isopods are the homogenisation of agricultural landscapes and the increased urbanisation in many parts of the world. Such habitats are subject to faunal homogenisation processes that favour a small number of adaptable, habitat generalist, isopod species. Even though we have not been able to document decreased levels of local (alpha) diversity in such habitats, overall diversity is negatively affected by decreasing beta-diversity among such localities. Nevertheless, this process has started to attract the attention of isopod researchers only recently, so we still need more data and also from a wider geographical range, before we can evaluate these processes reliably.

So, which are the most important open questions on the subject that should lead isopod research in the years to come? Herein, we give some answers to this question, but these should not be regarded as a finite list. In fact, we need input from a wide variety of research field in order to be able to draw a good picture of distribution patterns under environmental, including climate, change.

The first open question is both the most trivial and the most difficult to answer, but still is the most important: we need to record global isopod diversity, in terms of species occurrence and exact distribution ranges at a habitat and a regional scale, but also in terms of population abundance. How can we evaluate distribution patterns without a solid knowledge of actual distributions? Research on isopod distribution, of course, should be coupled with advancements in isopod taxonomy and phylogenetic analysis, using also molecular data, so that we can get a good picture of genetic diversity and variation patterns, in addition to a more robust natural taxonomy of isopods.
Global and regional latitudinal and altitudinal patterns of isopod diversity are still largely unknown. In addition, research on such gradients might also take into account possible trends in life-history parameters or other relevant features, such as body size etc. In addition, explorations of ‘species – area – habitat diversity’ relationships at different scales and in different systems (‘habitat islands’, oceanic versus continental islands etc.) could provide crucial information on processes that shape isopod communities.
Isopod invasions and long-distance dispersal remain a terra incognita for isopods, with current knowledge based mainly on anecdotal evidence. We need to solidly document such processes, possibly also using molecular approaches. There are several closely related questions that we also need to address, such as: What constitutes a propagule for different isopod species? To what extent are isopods able to exhibit niche shifts in order to establish populations in new habitats?
We need to employ state-of-the-art tools in order to predict distribution changes, taking advantage of all evidence available, at least for those species that have been studied in detail for many years in the past. Such tools include modern Species Distribution Models that are not solely based on species presence and/or absence data, but are also able to incorporate information on life-history parameters, abundance etc. There is a wide array of relevant models that are continuously improved and applied to a vast variety of taxa. We still lack, though, any application on terrestrial isopods, despite the fact that the information needed to apply some of these models is available, at least for a few, mostly European, regions.
We need detailed data on the life history (longevity, fecundity etc.) and other autecological parameters of as many species as possible. These data may also include tolerance to environmental factors, reaction norms of different phenotypes etc. Such data are crucial for evaluating population trends, niche shifts, for population viability analyses etc.
We need further research on expected changes of soil processes that might affect isopod distribution. Soil dynamics are under intensive research for quite a long time. The complexity of the systems, though, has not allowed researchers to provide sufficient models of soil processes under supposed climate change variations. The rapidly evolving tools of ‘next generation sequencing’, especially those concerning microbiome analysis, might prove of prime importance towards an increased understanding of a variety of relevant processes, especially taking into account the role of micro-organisms as food for isopod species. The effects of temperature increase and changes in local precipitation regimes on soil processes must be studied in detail, because they might play a crucial role in determining isopod distribution, acting synergistically with other factors, such as physiological, behavioural etc.
Functional diversity, including ‘ecomorphological strategies’, is tightly connected with species distribution dynamics. We urgently need a classification of isopod features, including aspects of morphology, physiology, behaviour, and several life-history parameters that will provide a meaningful description of functional diversity. Within this framework, it will be possible to make a finer analysis of isopod body plans, and to identify additional features that might be connected to these plans. The analysis of current functional diversity will offer lots of evidence for possible future trends under climate change.
We still lack robust data on isopod intra- and interspecific interactions. Available data are both taxonomically and geographically restricted, so we need to study such interactions for more species, coming also from more biomes. Interspecific interactions, of course, should not be restricted to isopods, but should also include possible interactions with predators, parasites, hosts, competitors and/or other species that might interact with isopods, such as other detritivores like diplopods etc. The examples of myrmecophilous species with the role of relations with ants for their distribution described in this chapter are highly indicative for the importance of this line of research for evaluating distribution dynamics under environmental change.
Research on evolutionary processes pertaining to isopod diversification is still lacking today, even though it is crucial in order to evaluate long-termed effects of current changes for isopod species. The role of environmental stresses as selective forces leading to speciation or extinction should be explored also within the framework of adaptive and non-adaptive radiations. The effects of population bottlenecks and population isolation due to habitat fragmentation should be closely studied in different species from a variety of isopod clades. We should also note here that evolutionary studies should particularly address the issue of regressive evolution, related to the path towards a life in caves or in deep soil, a path very commonly followed by isopods.
Last, but not least, we have to take full advantage of the unique features of terrestrial isopods among animal taxa, features that make them exceptionally useful models for the study of a wide range of phenomena, including those related to climate change effects on distribution. Isopods provide a unique opportunity for comparative studies among clades that represent almost all stages of evolution from a life in the sea to a life in almost all terrestrial habitats.
As a conclusion to this paper, we have to stress the need for an up to date, revised web-based isopod database, where all new data on species occurrences and, possibly also other data, will be regularly added in a format that will enable communication with GIS analytical tools etc.
